# Match Fatigue Time-Course Assessment Over Four Days: Usefulness of the Hooper Index and Heart Rate Variability in Professional Soccer Players

**DOI:** 10.3389/fphys.2019.00109

**Published:** 2019-02-19

**Authors:** Alireza Rabbani, Filipe Manuel Clemente, Mehdi Kargarfard, Karim Chamari

**Affiliations:** ^1^Department of Exercise Physiology, Faculty of Sport Sciences, University of Isfahan, Isfahan, Iran; ^2^Department of Medical and Performance, Sporting Clube de Portugal, Lisbon, Portugal; ^3^Escola Superior de Desporto e Lazer, Instituto Politécnico de Viana do Castelo, Melgaço, Portugal; ^4^Instituto de Telecomunicações, Covilhã, Portugal; ^5^Athlete Health and Performance Research Center Aspetar, Qatar Orthopaedic and Sports Medicine Hospital, Doha, Qatar

**Keywords:** soccer, monitoring, team sports, competition, association football

## Abstract

The aims of the present study were to (a) examine recovery time-course and (b) analyze the usefulness of the Hooper-Index (wellness index) and resting heart rate variability (HRV) in professional soccer players during an in-season phase. The Hooper-Index and resting HRV were collected on matchday and on the four following days in three consecutive in-season weeks in nine players (25.2 ± 4.3-years). The usefulness of monitoring variables was assessed by (a) comparing noise (typical error, TE) to the smallest worthwhile change (SWC) (TE/SWC) and (b) comparing match-related changes (i.e., signal) to TE (i.e., signal-to-noise ratio). Between-days standardized differences in the changes of Hooper-Index and HRV were compared to the SWC using magnitude-based inferences. The magnitudes of TE were small and moderate for the Hooper-Index and HRV, respectively. The Hooper-Index showed to be more useful than HRV for monitoring match-induced fatigue as having a lower TE/SWC (3.1 versus 4.4) and a higher signal-to-noise ratio (5.5 versus 1.5). Small-to-very large [range of effect sizes, 0.48; 2.43, confidence limits (0.22; 2.91)] and moderate-to-large [-1.71; -0.61 (-2.44; -0.03)] detrimental changes in Hooper-Index and HRV, respectively, were observed on the days following matchday. While group analyses showed a similar pattern for recovery time-course, more individual players responded, similarly when tracked using the Hooper -Index compared to when they were tracked using HRV. An inverse moderate within-individual relationship was observed between changes in the Hooper index and HRV [*r* = -0.41, (-0.60, 0.18)]. The Hooper index is an easy-to-use, no-cost, and non-invasive monitoring tool and seems promising for tracking match-induced fatigue during in the season in professional soccer.

## Introduction

Professional soccer players are exposed to spikes in training load arising from official matches during in-season phases (Thorpe et al., 2015, 2016b; Rabbani et al., 2018). Research has shown impairments in physiological and performance measures following high training load sessions with negative effects lasting from minutes to days (Barnett, 2006). These impairments, if not managed properly by the coaching staff by adjusting individual training loads and/or implementing specific recovery strategies, might result in excessive fatigue and overreaching (Brink et al., 2012; Thorpe et al., 2017). However, well-structured training plans that involve sensitive monitoring tools (objective/perceptive) lead to reduced risk of injury, increased player availability and a high chance of team success in high-level soccer (Akenhead and Nassis, 2016). Hence, the monitoring of athletes’ fatigue status and wellness is getting increasing attention both from the scientific community (Saw et al., 2015; Thorpe et al., 2015, 2016a,b, 2017; Djaoui et al., 2017) and practitioners working in the field (Akenhead and Nassis, 2016).

The attention given to the post-match recovery status of soccer players has been increasing recently (Thorpe et al., 2015, 2016a,b). Tracking the post-match recovery of soccer players requires monitoring tools that are sensitive to match-induced fatigue to help practitioners making decisions on a daily basis (Thorpe et al., 2016b). In this sense, only a few variables – including vagal-related time domain parameters of heart rate variability (HRV) and self-reported wellness measures – have shown promising results when used in elite soccer players (Thorpe et al., 2015). Furthermore, while post-match inflammatory and performance measures show a recovery time-course in soccer (Ispirlidis et al., 2008), it is unknown whether monitoring tools – including resting HRV and self-reported wellness measures – are able to detect the same tendency/pattern. However, the recovery time-course of these measures on days following official matches have not yet been examined in professional soccer players.

More specifically, the Hooper-Index and/or its subsets (i.e., sleep quality and the quantities of stress, fatigue, and muscle soreness) (Hooper and Mackinnon, 1995) have recently been shown as promising tools for monitoring fatigue in soccer players (Thorpe et al., 2015; Fessi et al., 2016; Rabbani and Buchheit, 2016; Clemente et al., 2017). The Hooper-Index, in particular, has been reported to be associated with training load in professional soccer players (Moalla et al., 2016). HRV has also been shown to be pertinent with daily fluctuations of training load in soccer players (Thorpe et al., 2015). There are, however, two main approaches for examining the usefulness (sensitivity) of a monitoring tool: (a) comparing the noise [typical error (TE)] of variables to the smallest worthwhile change (SWC) (Hopkins, 2004), and (b) comparing the magnitude of match-induced changes in variables with its TE (i.e., signal-to-noise ratio) (Buchheit, 2014). However, to the best of authors’ knowledge, no study has yet examined the usefulness of the Hooper-Index or resting HRV in professional soccer players. Information about the usefulness of monitoring variables would help practitioners choose the most appropriate tools to use in their daily practice for tracking soccer players’ general fatigue. Therefore, the aims of this study were to (a) examine recovery time-course and (b) compare the usefulness of the Hooper-Index and resting HRV in professional soccer players during an in-season phase.

## Materials and Methods

### Participants

Nine outfield soccer players (25.2 ± 4.3 years, 76.1 ± 5.7 kg, 179.2 ± 7.4 cm, and 23.7 ± 0.6 kg/m^2^ of body mass index) competing in the Iranian Premier League participated in this study. First-squad players were monitored in terms of self-reported wellness measures and resting HRV. This data was collected on matchday and on the 4 days immediately following matchday for three consecutive in-season weeks. The Ethics Committee of the Faculty of Sports Sciences of the University of Isfahan approved the study design. All players provided written informed consent before commencing the study.

### Study Design

Players performed three official matches (1 week apart) during a 3-week period. These three matches were weeks 3–5 of the first half of the 2015–2016 season. Participants who played for at least 70 min in all three selected matches were included in data analysis. The mean ± standard deviation (SD) of playing time for the players are 91.4 ± 7.2 min, respectively. Internal training load was computed for matchday (MD) and for the 3 days following MD (MD+1, MD+2, and MD+3) (see below). Subjective ratings of wellness and HRV were also collected on MD and on the 4 days following the MD in the morning just after individuals woke up (between 7:00 and 9:00 a.m.). The training sessions that took place on the 3 days following MD were similar during the selected period, as per the team coach’s normal routine. On MD+1, players attended a recovery session which included 20 min of light aerobic activity, 10 min of light static stretching, and 12 min of cold-water immersion (10–12°C). On MD+2, 60 min of technical and tactical training was prescribed. On MD+3, players performed a combined technical/tactical (65 min) and conditioning (25 min of small-sided games) session.

### Methodology

Players were habituated with all monitoring variables (i.e., internal training load, Hooper index, and HRV) during the pre-season phase. The rating of perceived exertion (RPE), based on Borg’s CR-10 scale, was collected from each participant within 30 min of the cessation of the training and match sessions. The rated RPE of the player was then multiplied by the time of the session/match (in minutes) to compute sRPE (Foster et al., 2001), which has been shown to be a valid indicator of internal training load in soccer (Impellizzeri et al., 2004). HRV and wellness measures were collected from all players before training and match sessions. A smartphone application (Elite HRV^TM^) and an HR monitor (Polar H7, Polar Electro Oy; Kempele, Finland) were used by each individual to measure HRV. A logarithm of the square root of the mean of the sum of the squares of differences between adjacent normal R-R intervals (Ln rMSSD) as a recommended HRV measure for monitoring athletes’ training status (Buchheit, 2014) reported by Elite HRV^TM^ was used for the analysis. Elite HRV^TM^ has been shown to be a valid application, as it has a large degree of agreement with the Kubios HRV analysis software for rMSSD determination (Perrotta et al., 2017). Players were instructed to record HRV in an upright sitting position with their smartphone after waking in the morning. HRV recordings lasted for 1 min, preceded by a 1-min stabilization period as they remained in their upright sitting position. All players were instructed to record HRV under spontaneous breathing to have consistency in the Ln rMSSD results (Saboul et al., 2013). A customized questionnaire comprising perceived sleep quality and quantities of stress, muscle fatigue, and muscle soreness based on the recommendations of Hooper and Mackinnon (1995) was used to collect data from the subjective self-reported wellness measures. Each question was scored on a 7-point scale on which “1” and “7” represented “very, very good” and “very, very poor” wellness ratings, respectively. The Hooper-Index, as an overall wellness measure, was computed by summing its four subsets (Moalla et al., 2016).

### Statistical Analysis

**FIGURE 1:**
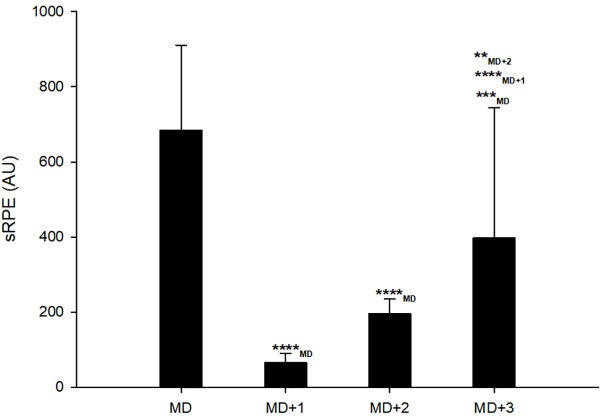
Session ratings of mean session rating of perceived exertion (sRPE) on matchday (MD) and days following it. ^∗∗^, ^∗∗∗^, and ^∗∗∗∗^ represent moderate, large, and very large effect size, respectively.

Data are presented with means and 90% confidence limits (CL) or SD in the text and tables where specified; means and SD are given in Figure 1, 2. Data were first log-transformed to reduce bias arising from non-uniformity error. Match-to-match variations in Hooper-Index and Ln rMSSD were analyzed using the typical error of measurement (TE) in standardized units (Cohen’s d principle) (Cohen, 1988) and expressed as a coefficient of variation (CV) (Hopkins, 2000b). Mean values of variables collected on MD+1 to MD+4 were used for analyzing recovery time-course. The usefulness of monitoring variables (Hooper-Index and Ln rMSSD) were assessed by (a) comparing their noise (TE) to the SWC and (b) comparing match-related changes (i.e., signal) with TE (i.e., signal-to-noise ratio) (Buchheit, 2014). Between-days standardized differences in the changes of Hooper Index data, Ln rMSSD and sRPE were compared to the smallest worthwhile change (SWC, 0.2 × between-participants SD) using magnitude-based inferences (Batterham and Hopkins, 2006). The Hopkins scale was used for magnitude interpretation: <0.2: trivial; >0.2-to-0.6: small; >0.6-to-1.2: moderate; >1.2: large (Hopkins et al., 2009). The probabilities were used to make a qualitative probabilistic mechanistic inference about the true effect with thresholds as follows: 25–75%: possible; >75-to-95%: likely; >95-to-99%: very likely; >99%: most likely (Hopkins et al., 2009). If the probability of the effect being positive and negative were both >5%, the effect was reported as unclear; otherwise, the effect was clear and reported as the magnitude of the observed value. Individual changes in monitoring variables on the days following MD (MD+1, MD+2, MD+3, and MD+4) were also assessed, using a specifically designed spreadsheet (Hopkins, 2000a) in which both TE and the SWC were considered. For individual analyses, only changes with probabilities higher than 75% were rated as substantial. A Pearson correlation coefficient was also used to measure within-individual associations between changes in Hooper-Index and Ln rMSSD. The correlation coefficient (r, 90%CL) was ranked as trivial (<0.10), small (>0.10-to-0.30), moderate (>0.30-to-0.50), large (>0.50-to-0.70), very large (>0.70-to-0.90), nearly perfect (>0.90-to-0.99), and perfect (1) (Hopkins et al., 2009).

**FIGURE 2:**
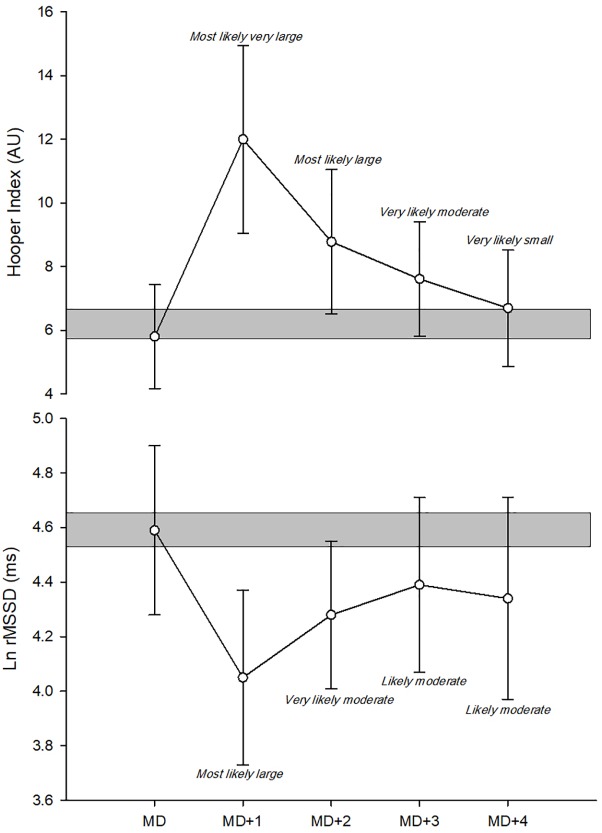
Recovery time-course of monitoring variables in group analyses. Ln rMSSD, logarithm of the square root of the mean of the sum of the squares of differences between adjacent normal R-R intervals; AU, arbitrary units.

## Results

### Match-to-Match Variations and Usefulness of the Hooper Index and Ln rMSSD

The magnitudes of TE were small and moderate for the Hooper-Index and Ln rMSSD, respectively (Table 1). The values of TE/SWC for the Hooper-Index and Ln rMSSD were 3.11 and 4.47, respectively. The signal-to-noise ratio of the Hooper-Index and Ln rMSSD were 5.54 and 1.55, respectively (Table 1).

**Table 1:** Reliability of subjective and objective monitoring variables.

Variable	Trial 1	Trial 2	Trial 3	All trials	TE (%, CV)	Standardized	SWC (%)
	Mean ± SD	Mean ± SD	Mean ± SD	Mean ± SD	(90% CL)	TE(90% CL)	(90% CL)
Ln rMSSD (ms)	4.55 (0.35)	4.59 (0.38)	4.64 (0.46)	4.59 (0.40)	7.6 (5.8; 11.9)	0.85 (0.66; 1.31)	1.7 (1.2; 3.0)
Hooper index (AU)	5.56 (1.67)	5.94 (2.19)	5.89 (1.54)	5.80 (1.82)	19.6 (14.8; 31.9)	0.59 (0.45; 0.91)	6.3 (4.5; 11.0)


*Ln rMSSD, logarithm of the square root of the mean of the sum of the squares of differences between adjacent normal R-R intervals; TE, typical error; SWC, smallest worthwhile change.*

### Recovery Time-Course of the Hooper Index and Ln rMSSD in Group Analyses

Very large decreases were observed in sRPE on MD+1 [-90.7%, 90% CL (-93.2; -87.4); standardized difference ES: -6.4 (-7.2; -5.5)] and on MD+2 [-70.6%, (-77.5; -61.5); ES: -3.3 (-4.0; -2.5)] compared with MD (Figure 1). A large decrease was also observed in sRPE on MD+3 [-49.9%, (-65.1; -28.1); ES: -1.86 (-2.84; -0.89)] compared with MD (Figure 1). However, MD+3 showed moderately and very largely higher sRPE compared with MD+2 [41.3%, (19.6; 57.1); ES: 0.86 (0.35; 1.37)] and MD+1 [81.4%, (89.0; 68.6); ES: 2.72 (3.57; 1.87)], respectively (Figure 1).

Most likely large and most likely very large detrimental changes were observed on MD+1 compared with MD for the Hooper-Index [108.7%, (80.2; 141.8); ES: 2.43 (1.94; 2.91)] and Ln rMSSD [-11.8%, (-16.4; -6.9); ES: -1.71 (-2.44; -0.98)], respectively (Figure 2). Most likely large and most likely very likely moderate detrimental changes were also observed on MD+1 compared with MD for the Hooper-Index [-51.9%, (31.9; 75.1); ES: 1.38 (0.91; 1.85)] and Ln rMSSD [-6.9%, (-10.4; -3.2); ES: -0.97 (-1.50; -0.44)], respectively (Figure 2). Very likely moderate and likely moderate detrimental changes were observed on MD+3 compared with MD for the Hooper-Index [32.5%, (14.3; 53.7); ES: 0.93 (0.44; 1.42)] and Ln rMSSD [-4.3%, (-8.3; -0.2); ES: -0.61 (-1.18; -0.03)], respectively (Figure 2). Very likely small and likely moderate detrimental changes were observed on MD+4 compared with MD for the Hooper-Index [15.8%, (6.9; 25.5); ES: 0.48 (0.22; 0.75)] and Ln rMSSD [-5.6%, (-11.6; 0.7); ES: -0.79 (-1.67; 0.09)], respectively (Figure 2).

### Recovery Time-Course of the Hooper Index and Ln rMSSD in Individual Analyses

**FIGURE 3:**
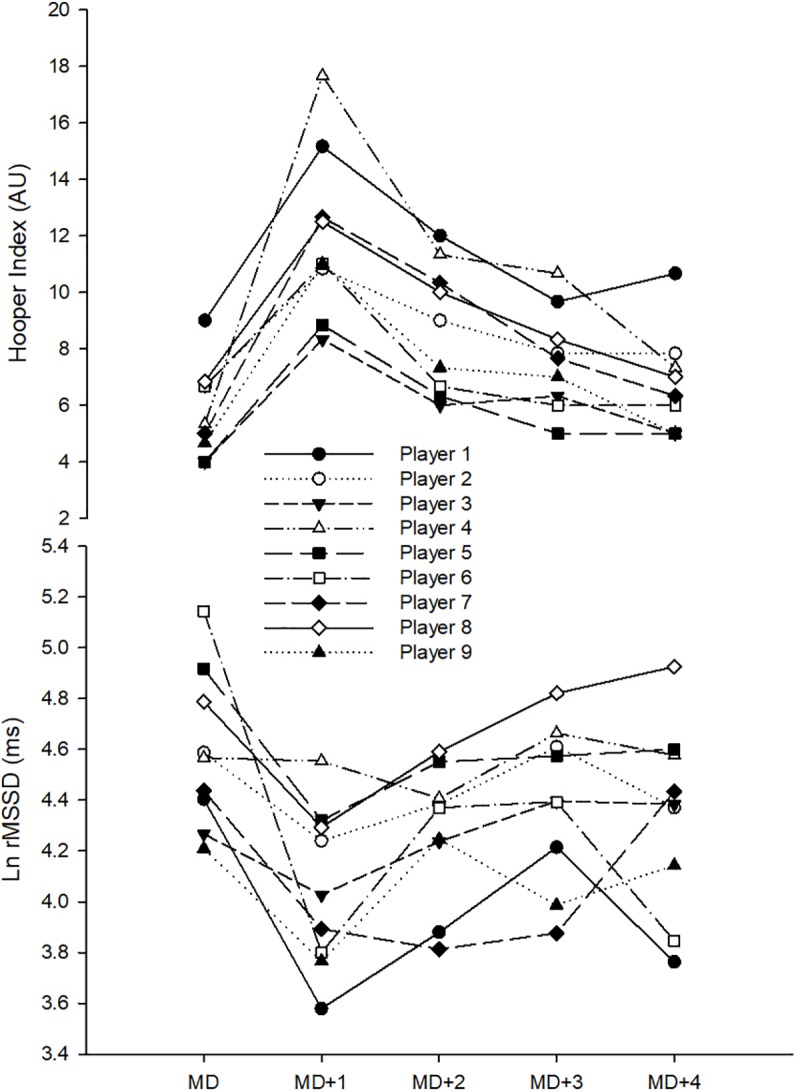
Recovery time-course of monitoring variables in individual analyses. Ln rMSSD, logarithm of the square root of the mean of the sum of the squares of differences between adjacent normal R-R intervals; AU, arbitrary units.

In individual analyses, while all players showed very likely to most likely substantial detrimental changes in the Hooper-Index (Table 2 and Figure 3) on MD+1 compared to MD, only 22% of them showed substantial detriments in Ln rMSSD (Table 3 and Figure 3). Comparing MD+2 to MD, 67% of players showed substantial detrimental changes in the Hooper-Index (Table 2 and Figure 3), but only 22% showed substantial detriments in Ln rMSSD (Table 3 and Figure 3). Comparing MD+3 to MD, 45% of players showed substantial detrimental changes in the Hooper-Index (Table 2 and Figure 3), but only 11% showed substantial detriments in Ln rMSSD (Table 3 and Figure 3). Comparing MD+4 to MD, only 11% of players showed a substantial difference in Ln rMSSD (Table 3 and Figure 3).

**Table 2:** Individual responses of soccer players to Hooper index questionnaire.

Player	MD + 1			MD + 2			MD + 3			MD + 4		
	% of	% of		% of	% of		% of	% of		% of	% of
	change	chances	Rating	change	chances	Rating	change	chances	Rating	change	chances	Rating
Player 1	-69%	2/2/96	Very likely	-33%	11/6/83	Unclear	-7%	33/12/55	Unclear	-19%	21/10/69	Unclear
Player 2	-63%	2/3/95	Very likely	-35%	9/8/83	Unclear	-18%	21/13/66	Unclear	-18%	21/13/66	Unclear
Player 3	-108%	0/1/99	Very likely	-50%	3/8/89	Likely	-58%	2/6/92	Likely	-25%	12/18/70	Unclear
Player 4	-231%	0/0/100	Most likely	-113%	0/0/99	Very likely	-100%	0/1/99	Very likely	-38%	7/9/83	Unclear
Player 5	-121%	0/0/99	Very likely	-58%	2/6/92	Likely	-25%	12/18/70	Unclear	-25%	12/18/70	Unclear
Player 6	-65%	2/3/95	Very likely	0%	42/16/42	Unclear	10%	58/15/27	Unclear	10%	58/15/27	Unclear
Player 7	-153%	0/0/100	Most likely	-107%	0/1/99	Very likely	-53%	3/5/91	Likely	-27%	12/14/74	Unclear
Player 8	-83%	1/1/98	Very likely	-46%	5/5/89	Likely	-22%	17/12/72	Unclear	-2%	38/16/46	Unclear
Player 9	-136%	0/0/100	Most likely	-57%	3/5/92	Likely	-50%	4/7/90	Likely	-7%	22/29/49	Unclear


*MD, matchday.*

**Table 3:** Individual responses of soccer players in Ln rMSSD.

Player	MD + 1			MD + 2			MD + 3			MD + 4		
	% of	% of		% of	% of		% of	% of		% of	% of
	change	chances	Rating	change	chances	Rating	change	chances	Rating	change	chances	Rating
Player 1	18.7%	88/11/1	Likely	11.9%	68/29/3	Possibly	4.3%	37/50/13	Unclear	14.5%	77/21/2	Likely
Player 2	7.6%	51/41/8	Unclear	4.4%	39/48/13	Unclear	-0.5%	22/52/25	Unclear	4.7%	40/48/12	Unclear
Player 3	5.6%	41/49/10	Unclear	0.7%	24/55/20	Unclear	-3.0%	15/54/31	Unclear	-2.7%	16/54/30	Unclear
Player 4	0.3%	25/53/23	Unclear	3.5%	35/50/15	Unclear	-2.1%	18/52/30	Unclear	-0.2%	23/53/24	Unclear
Player 5	12.1%	71/25/4	Possibly	7.5%	53/39/9	Unclear	7.0%	51/40/9	Unclear	6.4%	48/41/10	Unclear
Player 6	26.1%	98/2/0	Very Likely	15.0%	81/17/2	Likely	14.6%	80/18/2	Likely	25.2%	98/2/0	Very likely
Player 7	12.2%	69/28/3	Possibly	14.0%	76/22/2	Possibly	12.6%	70/27/3	Possibly	0.1%	23/54/23	Unclear
Player 8	10.3%	63/32/5	Possibly	4.1%	39/47/14	Unclear	-0.7%	23/51/27	Unclear	-2.9%	17/49/34	Unclear
Player 9	10.5%	61/35/4	Possibly	-0.9%	20/56/24	Unclear	5.3%	40/50/10	Unclear	1.6%	27/55/18	Unclear


*Ln rMSSD, logarithm of the square root of the mean of the sum of the squares of differences between adjacent normal R-R intervals; MD, matchday.*

### Within-Individual Relationships Between Changes in Hooper Index and Ln rMSSD

**FIGURE 4:**
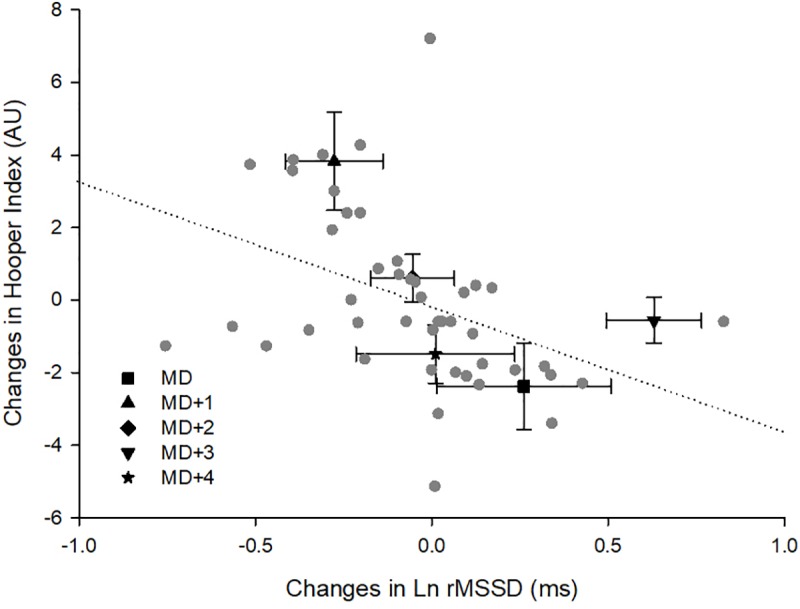
Within-individual relationships between changes of monitoring variables. Ln rMSSD, logarithm of the square root of the mean of the sum of the squares of differences between adjacent normal R-R intervals; AU, arbitrary units; MD, matchday.

Very large inverse within-individual correlations were observed between changes in the Hooper-Index and Ln rMSSD in five players [range *r* = -0.79 to -0.89, (-0.98; 0.08)]. An inverse nearly perfect within-individual association was also observed in one player between changes in the Hooper-Index and Ln rMSSD [*r* = -0.96, (-0.99; -0.63)]. However, inverse small [*r* = -0.16, (-0.86; 0.77)], moderate [*r* = -0.43; (-0.92; 0.61)], and large [*r* = -0.66, (-0.96; 0.36)] within-individual associations were observed in the three remaining players between changes in the Hooper-Index and Ln rMSSD. When all individual data were pooled together, an inverse moderate relationship was observed between changes in the Hooper-Index and Ln rMSSD [*r* = -0.41, (-0.60; -0.18)] (Figure 4). However, mean values of changes in Hooper-Index and Ln rMSSD showed an inverse nearly perfect relationship [*r* = 0.94, (-0.99; -0.51)] (Figure 4).

## Discussion

The purpose of this study was to examine the usefulness and recovery time-course of the Hooper-Index and HRV in professional soccer players during an in-season phase. The Hooper-Index was observed to be a more stable and sensitive measure than HRV when used to monitor match-induced fatigue. While group analyses showed a somewhat similar pattern in recovery time-course for both the Hooper-Index and HRV in the days following the match, individual players responded more, similarly when tracked with the Hooper-Index.

While the CV was lower for Ln rMSSD (7.6% cf. 19.6%), the Hooper -Index showed a lower standardized TE (0.59 cf. 0.85). The match-to-match variation in Ln rMSSD observed in this study is in agreement with a recent study reporting a CV of 7.6% (Nakamura et al., 2017) but lower than values reported in other studies in which values of 8% (Sinnreich et al., 1998) and up to 12.3% (Al Haddad et al., 2011) were found. Sinnreich et al. (1998) measured HRV on two occasions 2 months apart, which is considered long enough to influence autonomic regulation due to training-induced fatigue or adaptation (de Freitas et al., 2015; Nakamura et al., 2015; Flatt and Esco, 2016; Flatt et al., 2016). Different training statuses of participants studied by Al Haddad et al. (2011), when compared with our participants (moderately trained participants versus professional soccer players, respectively), might explain, at least in part, their greater TE. Therefore, the low magnitude of CV found in the present study might be associated with the training background of participants (highly trained team-sport athletes) similar to the study of Nakamura et al. (2017). Variability in Hooper-Index found here (19.6%) is higher than variations previously reported for its subsets, including sleep quality (13%), muscle soreness (9%), and fatigue (12%), in professional soccer players (Thorpe et al., 2015). However, it is obvious that the variations in Hooper-Index data computed by summing four different wellness subsets (i.e., sleep quality, stress, fatigue, and muscle soreness) result in higher variability. Nevertheless, to the best of authors’ knowledge, while some studies have compared differences or changes in Hooper-Index in soccer (Chamari et al., 2012; Haddad et al., 2013; Fessi et al., 2016; Moalla et al., 2016; Rabbani and Buchheit, 2016; Clemente et al., 2017) no study has reported its CV yet. That Ln rMSSD has a lower CV than the Hooper-Index in this study (7.6 versus 19.6) might suggest the greater effectiveness of the Ln rMSSD. Nevertheless, a lower TE in a monitoring tool does not necessarily mean it has greater usefulness *per se*. The usefulness (sensitivity) of a monitoring tool is usually determined by comparing its TE to a value deemed to be clinically or practically important (i.e., SWC, TE/SWC) (Jones et al., 2014) or dividing its signal (match-induced detrimental change in case of fatigue monitoring) by the TE (i.e., signal/noise ratio) (Buchheit, 2014). In this case, the result is that the Hooper ir-Index is more useful (sensitive) than Ln rMSSD, as it has a lower TE/SWC (3.1 versus 4.4) and a higher signal-to-noise ratio (5.5 versus 1.5).

The large-to-very large decreases observed here in sRPE from MD+1 to MD+3 (Figure 1) were not surprising and confirm the results of previous studies that showed significant reductions in internal (Thorpe et al., 2016b) and external (Thorpe et al., 2015) training loads on post-match days in professional soccer players. The magnitude of sRPE in the 3 days following the match tended to increase as time progressed, which suggests coaches try to gradually increase training load on the days (MD+2, MD+3) following the recovery session (MD+1) in high-level soccer. Moderately and very largely higher sRPE values observed on MD+3 compared with those observed on MD+2 and MD+1, respectively, might also suggest a load spike on MD+3. However, the coaching staff aimed to have the real training load spike occur on MD+4 during this observational period. Group analyses showed most likely very large and large sudden detrimental changes in the Hooper-Index and Ln rMSSD, respectively, on MD+1 compared to MD (Figure 2). However, on MD+2 and MD+3, a recovering tendency was observed both in Hooper index and Ln rMSSD data (Figure 2). On MD+4, only the Hooper-Index showed a better recovery status compared with MD+3 (Figure 2). These results show that both the Hooper-Index and Ln rMSSD can mirror the recovery time-course of the physiological status of soccer players following a match (Ispirlidis et al., 2008). Previous studies have shown post-match detrimental changes in the Hooper-Index or its subsets (Thorpe et al., 2015, 2016b; Rabbani and Buchheit, 2016) and Ln rMSSD (Thorpe et al., 2015), but the present study shows a recovery time-course pattern in these indices in professional soccer players. These results also suggest that soccer players were not fully recovered 4 days after a match; this finding is consistent with reported performance deteriorations 4 days post-match described elsewhere (Ispirlidis et al., 2008).

Individual analyses showed that, from MD+1 to MD+3, higher percentages of players showed detrimental changes to a greater extent in the Hooper-Index (45–100%) than in Ln rMSSD (11–22%). In other words, individual players responded more, similarly to the general recovery time-course pattern when tracked by the Hooper-Index than when tracked by Ln rMSSD. These results confirm previous evidence regarding different individual responses among athletes (Mann et al., 2014; Flatt et al., 2016; Rabbani et al., 2018) and suggest that the Hooper-Index is a more suitable tool for monitoring soccer players at an individual level.

While mean values of changes in the Hooper-Index and Ln rMSSD among individuals showed an inverse and nearly perfect relationship, an inverse but only moderate association was observed when all data were pooled together (Figure 4). Furthermore, although inverse very large to nearly perfect within-individual correlations were observed in six players, weaker relationships (small to moderate) were observed among the three remaining participants. These results may suggest that although strong general within-individual relationships exist between changes in the Hooper index and Ln rMSSD, all individuals may not show the same trend. Therefore, a detrimental change in the Hooper-Index is not always consistent with a detrimental change in Ln rMSSD at the individual level.

A better recovery time-course pattern was observed for the Hooper-Index both in group and individual analyses than for Ln rMSSD (Figure 2, 3), and this might suggest limitations when using HRV for tracking match-induced fatigue in soccer players. The Hooper-Index comprises different subsets and seems promising when monitoring soccer players (Moalla et al., 2016; Rabbani and Buchheit, 2016; Clemente et al., 2017), while resting Ln rMSSD can be influenced by cognitive activity and emotional state (Djaoui et al., 2017) and has been shown to be unable to track different levels of fatigue (Schmitt et al., 2015). The results, therefore, support the findings of Thorpe et al. (2016b) which showed that subsets of the Hooper-Index (sleep quality, fatigue, and muscle soreness) are clearly more sensitive than Ln rMSSD to daily fluctuations in training load in a standard in-season week. Therefore, the present study showed that the Hooper-Index is a more useful and sensitive tool than Ln rMSSD for monitoring match-induced fatigue in highly trained soccer players during the season. If the Hooper-Index and Ln rMSSD can mirror the post-match recovery time-course of such elite soccer players, the Hooper-Index is more suitable than HRV for tracking the fatigue status of this population (i.e., highly trained soccer players) during the season. However, this study is limited by the sample size and only data from nine players informed the study results. Therefore, further studies conducted on larger sample sizes with recruiting more number of players from different clubs are recommended to verify the conclusions. In this context, the authors of the present study would like to point out that finding a high number of highly trained elite soccer players is somewhat challenging, at least partly explaining the low number of participants of the present study.

## Author Contributions

AR, FC, and MK conceptualized the idea and participated in the design of the study. AR carried out the data collection and performed the statistical analysis. AR, FC, MK, and KC drafted the manuscript and revised critically for important intellectual content. All authors read and approved the final manuscript.

## Conflict of Interest Statement

The authors declare that the research was conducted in the absence of any commercial or financial relationships that could be construed as a potential conflict of interest.
